# Recovery of Butanol by Counter-Current Carbon Dioxide Fractionation with its Potential Application to Butanol Fermentation

**DOI:** 10.3390/ma9070530

**Published:** 2016-06-30

**Authors:** Miriam Solana, Nasib Qureshi, Alberto Bertucco, Fred Eller

**Affiliations:** 1 Department of Industrial Engineering DII, University of Padua, Via Marzolo 9, Padua 35131, Italy; alberto.bertucco@unipd.it; 2 United States Department of Agriculture (USDA), National Center for Agricultural Utilization Research (NCAUR), Functional Foods Research Unit, 1815 North University Street, Peoria, IL 61604, USA; Fred.Eller@ARS.USDA.GOV; 3 United States Department of Agriculture (USDA), National Center for Agricultural Utilization Research (NCAUR), Bioenergy Research Unit, 1815 North University Street, Peoria, IL 61604, USA

**Keywords:** butanol, CO_2_ fractionation, separation efficiency, butanol rich extract, raffinate

## Abstract

A counter-current CO_2_ fractionation method was applied as a mean to recover n-butanol and other compounds that are typically obtained from biobutanol fermentation broth from aqueous solutions. The influence of operating variables, such as solvent-to-feed ratio, temperature, pressure and feed solution composition was experimentally studied in terms of separation efficiency, butanol removal rate, total removal and butanol concentration in the extract at the end of the continuous cycle. With respect to the temperature and pressure conditions investigated, results show that the highest separation efficiency was obtained at 35 °C and 10.34 MPa. At these operating conditions, 92.3% of the butanol present in the feed solution was extracted, and a concentration of 787.5 g·L^−1^ of butanol in the extract was obtained, starting from a feed solution of 20 g·L^−1^. Selectivity was calculated from experimental data, concluding that our column performs much better than a single equilibrium stage. When adding ethanol and acetone to the feed solution, ethanol was detected in the water-rich fraction (raffinate), whereas the highest concentration of acetone was found in the butanol rich fraction (extract).

## 1. Introduction

The increase of oil prices and the depletion of fossil fuels have promoted the development of biofuels such as biobutanol and bioethanol [1]. Efforts to re-commercialize biobutanol are gaining remarkable attention; its potential to substitute for both ethanol and biodiesel in the biofuel market are estimated to be worth $247 billion by 2020 [2]. In addition, butanol is an important feedstock for the chemical industry, being used in the production of paint, solvents and plasticizers [3]. In this context, the optimization of the processes to produce and recover biobutanol is of utmost importance.

First of all, biobutanol needs to compete in cost (priced on an energy basis) with ethanol [4]. Biobutanol has a higher energy density, is less volatile, less explosive and less hygroscopic (does not pick up water) than ethanol. Moreover, butanol can easily mix with gasoline in any proportion, can be used in internal combustion engines, can be transported in existing pipe lines, and is less corrosive [5,6,7]. However, at present, improving the economics of butanol production is crucial for its re-commercialization and competition with ethanol [8].

Renewable butanol is produced from the fermentation of carbohydrates in a process often referred to as the acetone-butanol-ethanol (ABE) fermentation [4]. Butyric and acetic acids are first produced by *Clostridium acetobutylicum* or *C. beijerinckii* (acidogenesis), and in the subsequent phase (solventogenesis) butanol, acetone and ethanol are formed [8]. The concentration of butanol typically reached in the fermenter is rather low (10–20 g·L^−1^) because this compound is toxic to the butanol-producing microorganisms [9]. Distillation is traditionally carried out to recover butanol from the fermentation broth. However, its cost is high due to the low concentration of butanol and to the fact that water is the major component, with a boiling point below that of butanol (100 °C vs. 117.7 °C) [1].

A decrease in product recovery cost and purification from the dilute fermentation broth is included among the four recommendations to revive butanol fermentation and make it a commercially viable biofuel [10,11]. The methods so far proposed for the recovery of butanol include adsorption, liquid–liquid extraction, gas stripping, vacuum fermentation and pervaporation [8,12,13,14,15,16,17,18,19,20]. Conventional distillation method can still be improved but non-conventional methods are required to significantly reduce energy requirement and the associated cost [4].

The alternative we propose in this work consists of using counter-current CO_2_ fractionation to recover butanol from the fermentation broth. Carbon dioxide could be an appropriate solvent for the extraction of high molecular weight alcohols from aqueous solutions because high molecular weight alcohols, such as butanols, are less hydrophilic and less volatile than lower molecular weight alcohols. Further, the vapor pressure of butanol (bp = 117.7 °C) is significantly less than the vapor pressure of methanol or ethanol, making the separation of the solute from CO_2_ easier [21]. Moreover, CO_2_ is inert, non-toxic and can be easily recycled, resulting in an economic and environmental benefit. Counter-current operation facilitates the separation since it reduces the amount of solvent necessary, increases the throughput, and enables higher extract concentration in the solvent and lower residual concentrations in the raffinate than does single stage or multistage cross current operation [22].

To the best of our knowledge, only one study reports the recovery of butanol from aqueous solutions by using CO_2_ [21]. These authors reported that, using supercritical CO_2_ in a mechanically agitated extraction column at 10 MPa and 40 °C, approximately 0.02 wt % of butanol concentration in the raffinate and 85–90 wt % butanol in the extract can be obtained.

Our objective in this work was to investigate the effects of CO_2_ extraction parameters on the separation of butanol from aqueous solutions including solutions containing acetone and ethanol with the ultimate aim to apply this technology to ABE fermentation. In these studies, butanol removal by CO_2_ extraction is characterized by four important parameters known as separation efficiency, selectivity, rate of removal, and the concentration of butanol in the recovered product. Selectivity, concentration of butanol, and rate of removal presented here can be compared with other processes where butanol was recovered or removed. These processes include gas stripping, pervaporation, and vacuum fermentation. In the case of gas stripping and vacuum fermentation low selectivities, usually less than 20, are obtained.

## 2. Results

Separation efficiency was calculated as follows:

(1) $$\text{Separation efficiency}=\frac{X/\left(1-X\right)}{Z/\left(1-Z\right)}$$

where *X* = weight fraction of butanol in the extract, and *Z* = average of weight fraction of butanol in the raffinate and in the feed.

Removal rate was calculated using the following equation:

(2) $$\text{Removal rate}\;\left({\text{kg}}_{\text{butanol}\;}h^{-1}\right)=\left(\text{Feed}\;conc.\;-\text{Raffinate}\;conc.\;\right)\times \text{Feed flow rate}$$

Specific removal, i.e., the removal per unit of solvent used, was calculated as:

(3) $$\text{Specific removal}\;\left({\text{kg}}_{\text{butanol }}/{\text{kg}}_{{\text{CO}}_{2}}\right)=\frac{\text{Removal rate}}{{\text{CO}}_{2}\;\text{flow rate}}$$

CO_2_ space velocity was evaluated by:

(4) $${\text{CO}}_{2}\;\text{space velocity}\;\left({\text{min}}^{-1}\right)=\frac{{\text{CO}}_{2}\;\text{flow rate}\;}{\text{Volume of extractor}}$$

The total removal of butanol with respect to the feed solution at the end of the continuous cycle was calculated as:

(5) $$\text{Total removal}\;\left(\%\right)=\left(1-\frac{\text{Raffinate concentration}\;}{\text{Feed concentration}\;}\right)\times 100$$

Overall selectivity of butanol in the separation unit was evaluated by:

(6) $$\text{Selectivity}=\frac{X/\left(1-X\right)}{Y/\left(1-Y\right)}$$

where *X* = fraction of butanol in the extract, and *Y* = fraction of butanol in the raffinate (note that the same result is obtained when calculating selectivity from weight or molar fractions).

### 2.1. Effect of Solvent-to-Feed Ratio

Solvent-to-feed volume ratio (based on the flow rate) is an important parameter that affects the yield and the economics of the fractionation process. The influence of solvent-to-feed was studied at constant pressure (10.34 MPa) and temperature (25 °C) with ratios at 1.25, 2.5, 5 and 7.5. The average concentration of butanol in the feed solution was 20.1 ± 1.0 g·L^−1^. Separation efficiency, removal rate and total removal were calculated from the analysis measures.

**Figure 1:**
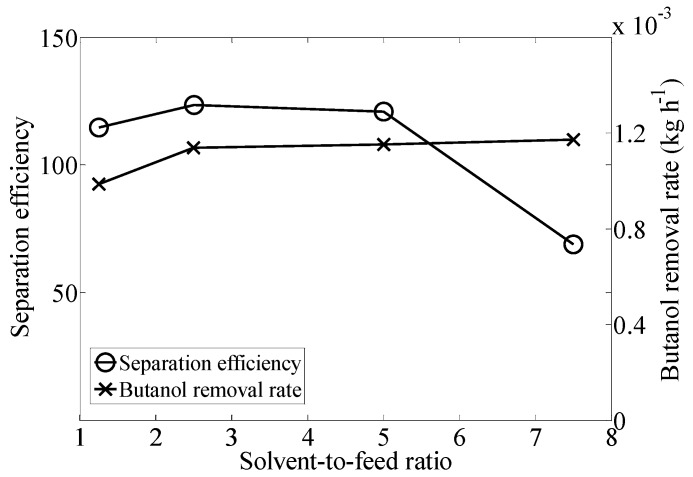
Effect of solvent-to-feed ratio on separation efficiency and butanol removal rate (kg·h^−1^) at constant pressure (10.34 MPa) and temperature (25 °C).

As illustrated in Figure 1, the effect of solvent-to-feed ratio between 1.5 and 5 on separation efficiency is not very significant. However, when the solvent-to-feed ratio was increased from 5 to 7.5, the efficiency decreased from 120.8 to 69.0. At a solvent-to-feed raio of 7.5, there may have been insufficient contact time to the butanol to partition into the CO_2_. The removal rate increased from 0.0010 to 0.0011 kg·h^−1^ when the solvent-to-feed-ratio was changed from 1.25 to 2.5, and it slightly increased when the flow rate was increased from 2.5 to 7.5.

The highest butanol concentration in the extract (603.2 g·L^−1^) was obtained at a solvent-to-feed ratio of 1.25 and it decreased as the solvent-to-feed ratio increased, as shown in Table 1. At the highest CO_2_ flow rate, more water is extracted leading to a lower concentration of butanol. Nevertheless, the total removal of butanol from the feed solution was lower at 1.25 than at higher solvent-to-feed ratio. From 2.5 to 7.5, the total removal increased slightly.

Considering the results presented in this section, a solvent-to-feed ratio of 2.5 was chosen to perform the experiments reported in the following sections. This result is in agreement with the work reported by Laitinen et al. [21], who used a solvent-to-feed ratio of 2.7.

**Figure 2:**
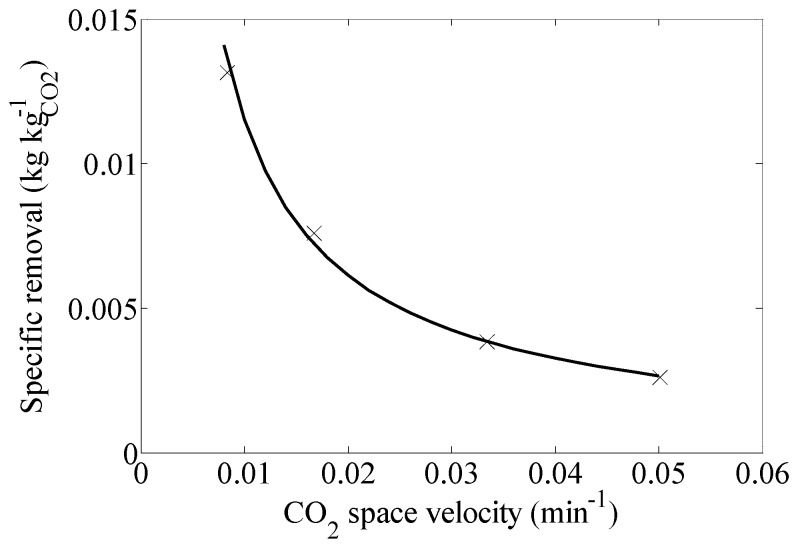
Effect of CO_2_ space velocity on specific removal (kg_butanol_·kg_CO_2__^−1^) at constant pressure (10.34 MPa) and temperature (25 °C).

Figure 2 shows the influence of the CO_2_ space velocity (min^−1^) on the specific removal of butanol (kg_butanol_·kg_CO_2__^−1^) considering the CO_2_ consumption. As expected, the higher the CO_2_ space velocity, the lower the removal of butanol for the same values of CO_2_ consumption.

### 2.2. Effect of Pressure and Temperature

Studies of pressure and temperature effects of CO_2_ processes are essential, since the solvating power of CO_2_ can vary significantly when these operating variables are changed. On the one hand, two tests were performed keeping constant pressure (10.34 MPa) and varying the temperature from 25 °C (liquid CO_2_) to 35 °C (supercritical CO_2_). On the other hand, results obtained at constant density of CO_2_ (0.842 g·mL^−1^) and diverse temperature and pressure are analyzed. In the latter, liquid CO_2_ at 25 °C and 10.34 MPa versus supercritical CO_2_ at 50 °C and 25.16 MPa were used. In all the runs, CO_2_ flow rate was kept constant at 2.5 mL·min^−1^ and the feed solution was pumped at 1 mL·min^−1^. The average concentration of butanol in water in the feed solution was 20.0 ± 1.0 g·L^−1^.

Looking at Table 2, it can be seen that there are no significant differences on the values of removal rate either when temperature is increased or when both temperature and pressure are changed. It is noteworthy that the separation efficiency was much higher in the experiment performed at 35 °C (351.5). This can be due to the higher temperature with respect to 25 °C or to the lower density with respect to the other two tests.

With regards to the total removal of butanol from the feed solution at the end of the continuous cycle, values in the range 92.3%–96.9% were obtained. At the highest temperature and pressure, 50 °C and 25.16 MPa, the removal value was slightly higher. The highest concentration of butanol in the extract was 787.5 g·L^−1^, obtained at 35 °C and 10.34 MPa.

Based on these results, it can be concluded that supercritical CO_2_ at 35 °C and 10.34 MPa is more efficient to recover butanol than liquid CO_2_. Increasing pressure and temperature up to 50 °C and 25.16 MPa, the total removal is slightly increased, but the separation efficiency is much lower than at 35 °C and 10.34 MPa.

### 2.3. Butanol Concentration

The concentration of butanol in the fermentation broth of batch reactors can vary from 12 to 20 g·L^−1^ depending on microbial strain and fermentation conditions [23,24,25,26]. In the effluents of immobilized cell continuous reactors [27] and integrated reactors where product is recovered simultaneously [28], it can be lower than 12 g·L^−1^. In a recent work [28], the concentration of biobutanol produced from corn stover varied from 3.2 to 9.1 g·L^−1^ when using different conditions. Hence, it is important to study a wide range of butanol concentrations in the feed solution and their influence on butanol recovery. For this purpose, experiments with butanol concentrations in the range 6.7–74.9 g·L^−1^ were performed. Although 74.9 g·L^−1^ butanol concentration is never reached in butanol bioreactors, this concentration is often obtained in the recovered aqueous phase in the product recovery experiments. Butanol separation from this aqueous phase is essential and it results in high product recovery rates. Recycle of this phase has been discussed below. In all the runs, the operating parameters were 10.34 MPa, 25 °C, 2.5 mL·min^−1^ of CO_2_ and 1 mL·min^−1^ of feed solution.

**Figure 3:**
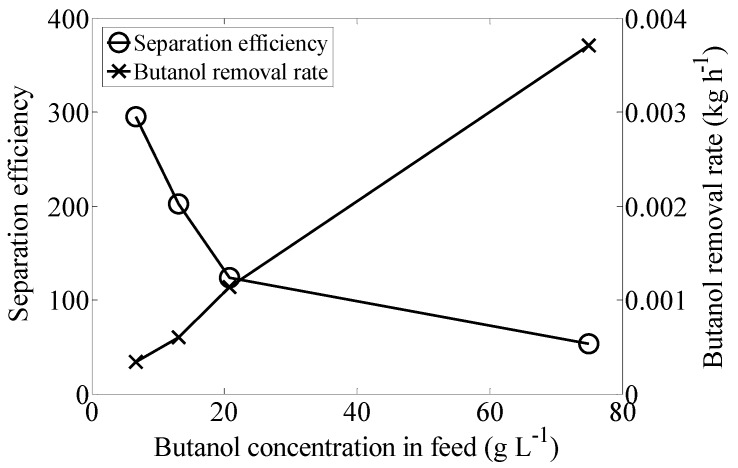
Influence of butanol concentration in feed (g·L^−1^) on separation efficiency and removal rate of butanol (kg·h^−1^). Experiments were performed at 25 °C and 10.34 MPa.

Results, represented in Figure 3, show that the lower the butanol concentration in the feed solution, the higher the separation efficiency. This result suggests that counter current CO_2_ fractionation would be especially effective when low concentrations of butanol are obtained in the fermentation broth. As predicted, the removal rate increased at higher concentrations of butanol.

As shown in Table 3, starting from a solution of 74.9 g·L^−1^, a concentration of 712.5 g·L^−1^ of butanol was obtained in the extract, removing 85.6% of the butanol present in the feed solution. However, the butanol concentration in the extract and the total removal rate did not follow a clear trend. Butanol removal rate increases essentially linearly with butanol concentration in the feed. However, separation efficiency decreased asymptotically as butanol concentration in the feed increased. This may be due to the highest butanol concentration tested being beyond the capacity of the CO_2_ (under the conditions of the solvent to feed ratio and column length used) to effectively remove the butanol from the fed.

### 2.4. Comparison with Equilibrium Data

**Figure 4:**
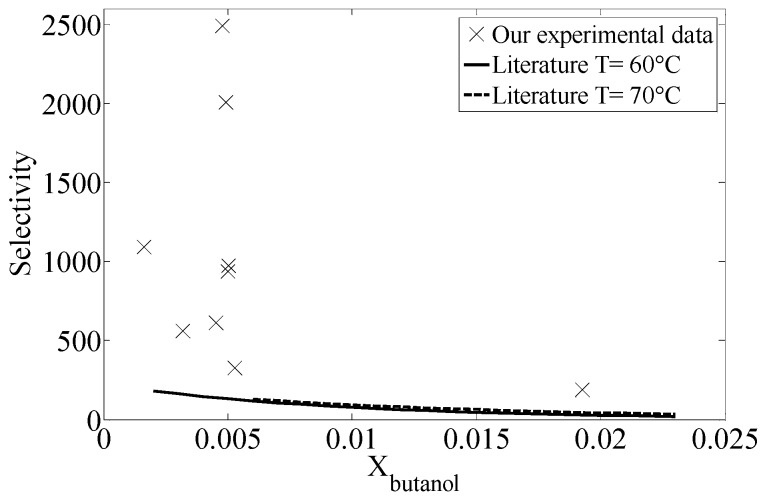
System CO_2_-water-butanol: selectivity of butanol calculated from reported data (60 and 70 °C) and comparison with the selectivity calculated from our experimental results.

The values of butanol selectivity calculated from the results presented above and the phase equilibrium data of the system CO_2_-water-butanol reported in literature [29] are illustrated in Figure 4. According to the data reported by these authors, our column clearly performs much better than a single equilibrium stage. In other words, the number of ideal separation stages provided by the column is consistently higher.

### 2.5. Butanol, Acetone and Ethanol

As mentioned above, acetone and ethanol are also formed in fermentation broth when biobutanol is produced. In the work published by Qureshi et al. [28], concentrations of acetone in a range 1.90–11.58 g·L^−1^ and concentrations of ethanol from 0.24 to 1.11 g·L^−1^ are reported. The aim of the study presented in this section was to find out if the major proportion of these compounds would be separated from butanol or would be obtained in the extract. The experiments were carried out at 10.34 MPa, 35 °C, 2.5 mL·min^−1^ CO_2_ and 1 mL·min^−1^ feed rate.

Table 4 shows the concentrations of the three compounds measured in the feed, raffinate and extract. In the experiment starting from the mixture of butanol and acetone, these two compounds were mostly collected in the extract flask, at concentrations of 740.7 and 24.0 g·L^−1^, respectively. Only 1.74 g·L^−1^ of butanol and 1.14 g·L^−1^ of acetone were found in the raffinate. However, when ethanol was added to the feed solution, traces of it were measured in the extract. Additionally, it should be considered that there were some losses due to material being adsorbed inside the column, especially the packing material with its high surface area, and during CO_2_ expansion where the compounds may be entrained with the CO_2_ vapor. This would be especially true for the compounds with higher vapor pressures (acetone > ethanol > propanol).

It should be noted that, in all the runs performed in this study, the extract collected presented two phases, the aqueous and the organic (butanol). Prior to the analysis, the solution was mixed until the two phases were combined in order to determine the total concentration of butanol in the extract without any loss of accuracy. Consequently, in large scale recovery units the organic phase (top layer) would be easily decanted off or separated. This phase contains small amount of water and can be dehydrated by removing it using molecular sieves such as silicalite. The aqueous phase contains approximately 78 g·L^−1^ butanol which can be recycled to the separation unit for further concentration.

## 3. Discussion

There are a number of reactor types which can be used to produce butanol including: (i) free or suspended cell batch reactors [23]; (ii) immobilized cell continuous reactors [27,30]; and (iii) membrane cell recycle reactors [31]. In suspended cell batch reactors butanol productivities of the order of 0.50 g·L^−1^·h^−1^ or less are achieved [26]. Application of CO_2_ extraction to remove butanol from these reactors would require much smaller recovery units as removal rates of butanol that were observed in the present studies were of the order of 8–9 g·L^−1^·h^−1^. The reactor productivities in most immobilized cell continuous reactors and membrane cell recycle reactors are of the order of 6.5 g·L^−1^·h^−1^ [27,30]. These high productivity reactors can also be integrated with the CO_2_ extraction process simply due to high rates of butanol removal. In some cases, reactor productivities over 15 g·L^−1^·h^−1^ have been reported [32]; however, these can also be integrated with butanol recovery by CO_2_ extraction. In such a case, the overall capital and operational costs would still be lower as compared to the systems which offer low productivities.

As mentioned in the introduction section of this article, the methods that have been studied for butanol removal from fermentation broths include adsorption, gas stripping, liquid–liquid extraction, vacuum fermentation, and pervaporation. Among these methods, vacuum fermentation and pervaporation appear to be promising. However, in the present studies on CO_2_ extraction, superior butanol separation efficiencies (351.5) than pervaporation (209; [33]) have been achieved. Also, the butanol concentration in the range of 787.5–829 g·L^−1^ have been obtained while using CO_2_ extraction process. These values are greater than the butanol concentration obtained employing pervaporation. Additionally, pervaporation requires membranes which are costly and have limited working life. With this information, it is clear that CO_2_ fractionation has a number of advantages over other product removal techniques.

In butanol fermentation, production of acetone and ethanol are also associated. In some cases, the ratios of acetone:butanol:ethanol are 3:6:1; however, this is not always the case. In the present studies, the ABE concentrations that we used were 4.71–6.37, 19.34–20.09, and 0.00–1.01 g·L^−1^, respectively (Table 4). While these concentrations are not in the ratio of 3:6:1, they represent the products we often obtain in our reactors using *C. beijerinckii* P260. The reader is advised that the ratios of these products vary from run to run and depends on a number of factors including feedstock/substrate used, fermentation conditions, presence of adequate/inadequate amounts of nutrients, and headspace gaseous pressure. It should be noted that different ratios of the fermentation products are expected to result in some variation of separation efficiencies, however, this is expected to be minimal.

These studies, conducted with model solutions, are encouraging and it is expected that this separation technique can be applied to recover ABE from actual fermentation broth which contains residual sugars, residual nutrients, microbial cells, polysaccharide (produced during fermentation), and organic acids such as acetic and butyric acids. At this stage, it is not clear whether it would be essential to remove all these fermentation components prior to ABE recovery. If removal of these products becomes essential, it would be more appropriate to apply steam stripping [34] to the fermentation broth which would strip ABE from this mixture leaving behind sugars, cells, polysaccharide, nutrients, and perhaps acids. The stripped product would mainly contain ABE which would be separated and concentrated using CO_2_ fractionation.

## 4. Materials and Methods

### 4.1. Materials

Butanol, acetone and ethanol were supplied by Fisher scientific (Fair Lawn, NJ, USA). Carbon dioxide was provided by ILL-MO products Co. (Jacksonville, IL, USA). Analytical grade acetone, butanol, ethanol, and n-propanol were obtained from Sigma Chemicals (St. Louis, MO, USA).

### 4.2. Fractionation Column

**Figure 5:**
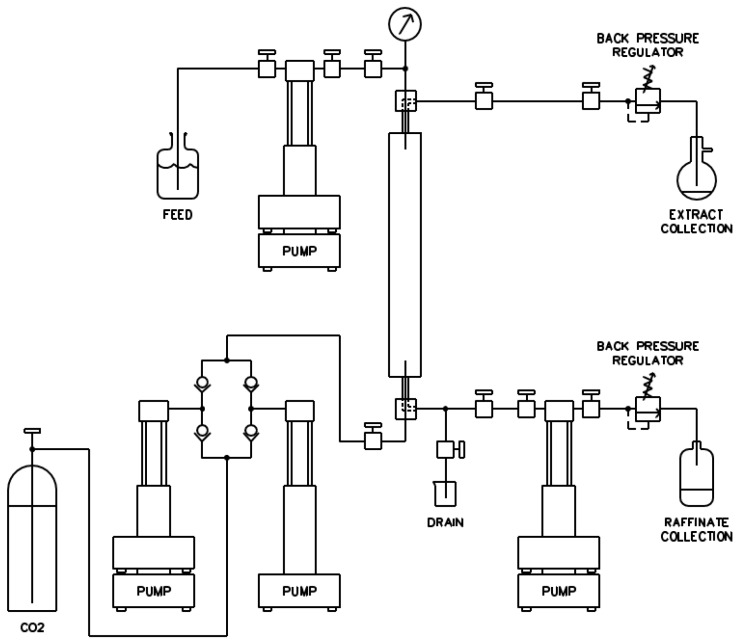
A schematic of CO_2_ counter-current fractionation system for recovery of butanol.

The basic design of the counter-current fractionation laboratory scale unit has been described elsewhere [35,36]. In this work, the gas booster pump was substituted by a syringe pump. The column was packed with seventy six vertically-stacked packing Pall Ring pieces, 316-stainless steel, of 0.016 m diameter (AMACS Process Tower Internals, Houston, TX, USA). A schematic of the complete apparatus used for this study is shown in Figure 5.

Initially, the system was pressurized and the column was heated at the experimental pressure and temperature. When equilibrium was reached, the feed solution was pumped at 1 mL·min^−1^ and the extract collection was started. The extract flask was submerged in dry ice to avoid the losses of butanol (or acetone and ethanol when applicable) by evaporation. CO_2_ entered from the bottom of the column and the butanol aqueous solution was fed from the top, so as to allow counter current contact of CO_2_ with the feed solution. Experiments lasted 300 min. Continuous feed solution flow was maintained for 200 min (continuous cycle), whereas only CO_2_ was pumped during the last 100 min. The raffinate was accumulated in the reservoir pump and was drained every 100 min intervals. One sample of the extract was collected after 200 min, namely at the end of the continuous cycle.

Tests at 10.34 and 25.16 MPa were performed. Temperature was varied in a range between 25 and 50 °C. Considering that CO_2_ critical temperature and pressure are 31.1 °C and 7.39 MPa, respectively, for the experiments performed at 25 °C, CO_2_ was at liquid state, whereas for the tests at 35 and 50 °C it was at supercritical conditions. CO_2_ flow rate was varied from 1.25 to 7.5 mL·min^−1^. The average concentration of butanol in the feed solution was 20.1 ± 1 g·L^−1^, except in the experiments presented in Section 2.3, where the effect of butanol concentration is analysed.

Each experimental run was replicated twice. The average and the standard deviation were calculated on the basis of the measures of the two tests.

### 4.3. Analytical Method

The compositions of extract, raffinate and feed were determined by gas chromatography as reported elsewhere [16,37].

## 5. Conclusions

In this work, the recovery of butanol from aqueous solutions by counter-current CO_2_ fractionation was studied. Results show that the effect of solvent-to-feed ratio on separation efficiency and removal rate is significant, obtaining the highest separation efficiency at 2.5. The specific removal (kg_butanol_·kg_CO_2__^−1^) decreased when increasing the CO_2_ space velocity. When studying the effect of pressure and temperature, the highest separation efficiency was obtained at 35 °C and 10.34 MPa, with a butanol concentration of 787.5 g·L^−1^ in the extract. At these operating conditions, 92.3% of the butanol present in the feed solution was removed. Different concentrations of butanol in the feed solution were tested, concluding that the higher the concentration of butanol in the feed solution, the higher the removal rate but the lower the separation efficiency. The comparison of phase equilibrium data of the system CO_2_-water-butanol with the experimental data presented in this work showed that our column provides a consistent number of ideal separation stages. Experiments with the other compounds typically obtained in the ABE process were also performed. Ethanol was collected in the raffinate, whereas the highest concentration of acetone was obtained in the butanol-rich fraction (the extract). The results obtained in this study form the basis to consider counter-current CO_2_ as an alternative method to recover butanol from butanol or acetone-butanol-ethanol (ABE) fermentation broths.

## Figures and Tables

**Table 1:** Effect of solvent-to-feed ratio on butanol concentration in extract (g·L^−1^) and total removal (%) after the continuous cycle.

Solvent to Feed Rate	Butanol Concentration in Extract (g·L^−1^)	Total Removal (%)
1.25	603.15 ± 13.22	76.87 ± 7.72
2.5	571.15 ± 42.21	93.01 ± 0.79
5	548.00 ± 82.80	94.01 ± 2.83
7.5	423.06 ± 39.26	96.14 ± 0.17

Values are mean ± S.D of duplicate experiments.

**Table 2:** Separation efficiency, removal rate (kg·h^−1^), concentration in extract (g·L^−1^) and total removal (%) using liquid CO_2_ (25 °C and 10.34 MPa) and supercritical CO_2_ (50 °C and 25.16 MPa).

Temperature (°C)	Pressure (MPa)	Separation Efficiency	Removal Rate (kg·h^−1^)	Butanol Concentration in Extract (g·L^−1^)	Total Removal (%)
25	10.34	123.49 ± 34.34	0.0011 ± 0.0002	571.15 ± 42.21	93.01 ± 0.79
35	10.34	351.48 ± 5.74	0.0011 ± 2.04 × 10^−5^	787.50 ± 3.54	92.33 ± 0.71
50	25.16	119.15 ± 6.09	0.0012 ± 4.16 × 10^−5^	552.4 ± 17.11	96.90 ± 0.90

Values are mean ± S.D of duplicate experiments.

**Table 3:** Butanol concentration in extract (g·L^−1^) and total removal (%) at different concentrations of butanol in the feed solution.

Butanol Concentration in Feed (g·L^−1^)	Butanol Concentration in Extract (g·L^−1^)	Total Removal (%)
6.7 ± 0.6	526.99 ± 58.28	84.46 ± 2.38
13.1 ± 0.7	609.2 ± 48.01	77.15 ± 1.50
20.8 ± 1.1	571.15 ± 42.21	93.01 ± 0.79
74.9 ± 5.3	712.5 ± 80.26	85.63 ± 4.93

Values are mean ± S.D of duplicate experiments.

**Table 4:** Concentration of butanol, acetone and ethanol (g·L^−1^) in the feed, raffinate and extract samples collected after the continuous cycle at 35 °C and 10.34 MPa.

Concentration in Feed (g·L^−1^)			Concentration in Raffinate (g·L^−1^)			Concentration in Extract (g·L^−1^)		
Butanol	Acetone	Ethanol	Butanol	Acetone	Ethanol	Butanol	Acetone	Ethanol
19.83 ± 2.83	-	-	1.49 ± 0.12	-	-	787.50 ± 3.54	-	-
20.09 ± 1.48	6.37 ± 1.37	-	1.74 ± 0.15	1.14 ± 0.11	-	740.73 ± 10.47	24.01 ± 1.34	-
19.34 ± 0.99	4.71 ± 0.38	1.01 ± 0.07	1.61 ± 0.08	0.57 ± 0.04	0.75 ± 0.01	829 ± 85	69.09 ± 0.81	0.00 ± 0.00

Values are mean ± S.D of duplicate experiments.
